# Antimicrobial Chitosan Conjugates: Current Synthetic Strategies and Potential Applications

**DOI:** 10.3390/ijms21020499

**Published:** 2020-01-13

**Authors:** Yukun Qin, Pengcheng Li

**Affiliations:** 1 Key Laboratory of Experimental Marine Biology, Center for Ocean Mega-Science, Institute of Oceanology, Chinese Academy of Sciences, Qingdao 266071, China; 2 Laboratory for Marine Drugs and Bioproducts, Pilot National Laboratory for Marine Science and Technology (Qingdao), No. 1 Wenhai Road, Qingdao 266237, China

**Keywords:** chitosan, conjugates, synthetic approach, antimicrobial activity, applications

## Abstract

As a natural polysaccharide, chitosan possesses good biocompatibility, biodegradability and biosafety. Its hydroxyl and amino groups make it an ideal carrier material in the construction of polymer-drug conjugates. In recent years, various synthetic strategies have been used to couple chitosan with active substances to obtain conjugates with diverse structures and unique functions. In particular, chitosan conjugates with antimicrobial activity have shown great application prospects in the fields of medicine, food, and agriculture in recent years. Hence, we will place substantial emphasis on the synthetic approaches for preparing chitosan conjugates and their antimicrobial applications, which are not well summarized. Meanwhile, the challenges, limitations, and prospects of antimicrobial chitosan conjugates are described and discussed.

## 1. Introduction

Chitosan, a product of the partial deacetylation of chitin, is a natural cationic linear polysaccharide. It has been recognized as one of the most promising renewable biopolymers due to its nontoxic, biodegradable, and biocompatible properties [1,2,3,4]. Chitosan possesses multiple activities such as antimicrobial, anti-oxidation, antiviral, and antitumor activities, among which its antimicrobial activities have attracted much attention in recent years [5,6,7,8]. It has been well documented that chitosan exhibits broad-spectrum antimicrobial activity [9,10]. It can inhibit the growth of a variety of fungi, bacteria and yeast [11]. Therefore, chitosan has broad application prospects in the fields of medicine, food, agriculture and so on [12,13].

However, the relatively poor antimicrobial properties of chitosan, as well as its low solubility in physiological environments, hinder its practical application [4,14]. In view of chitosan as an amino polysaccharide, the presence of reactive amino groups and hydroxyl groups makes it easy to chemically modify. Consequently, in recent years, many efforts have been made to functionalize chitosan to improve both its solubility and activity [15,16,17,18]. Extensive studies have proved that structural modification strategies such as quaternization [19], carboxylation [20], alkylation [21,22], and biologically active molecule conjugation [23,24] are very effective methods for obtaining more desirable chitosan derivatives.

One of the most attractive modification strategies is the attachment of bioactive substances to chitosan via covalent bonds. Such conjugation may maintain the fundamental properties of chitosan, enhance its solubility, and endow it with new properties ascribed to small active molecules [25,26]. In fact, polymer-drug conjugates have long been proven to be an effective form for improving the therapeutic effect and biological effect of a given drug [27,28,29], mainly in the following: (a). The hydrophobic drug is combined with the hydrophilic polymer to significantly improve the stability of the aqueous solution of the drug; (b). Triggering drug release for drug delivery; (c). Improving drug bioavailability and body fluid circulation time; (d). Avoiding drug degradation failure. According to the polymer conjugate model proposed by Ringsdorf, a chemical bond or tether formed by responses to a stimulus (pH, temperature, enzyme, etc.) is attached to the polymer backbone—if necessary, a targeting group or another group that alters the physical properties of the drug is introduced [30].

For chitosan conjugates, the basic composition of the complete chitosan-based conjugate system mainly includes active molecules (small molecule drugs, natural compounds, proteins/peptides, nucleic acids, etc.), chitosan carriers, coupled bonds or tethers. Sometimes, a solubilizing moiety or linker is needed. Based on this conjugation strategy, active ingredients such as caffeic acid [31], ferulic acid [32], tannic acid [33], catechin [34], curcumin [35], eurycomanone [36], streptomycin [37], gibberellin [38], cysteine [39], lysozyme [40], levofloxacin [41], cefuroxime [42] were reported to succeed in coupling with chitosan.

In the past decade, there have been extensive research reports on polysaccharide conjugates, and there have been some good review articles [43,44]. However, specifically regarding antimicrobial chitosan conjugates, there are currently very few review articles. It is obvious that the current covalent methodologies for chitosan conjugates and their potential applications in antimicrobial properties have not been well concluded and summarized.

Hence, this article will place substantial emphasis on the conjugation approach of chitosan conjugates and their antimicrobial applications, which have not been covered in the past 10 years. At the same time, we will also describe and forecast the challenges and problems in the current research and application of chitosan conjugates.

## 2. Methodologies for Covalent Bioactive Substances

**Figure 1:**
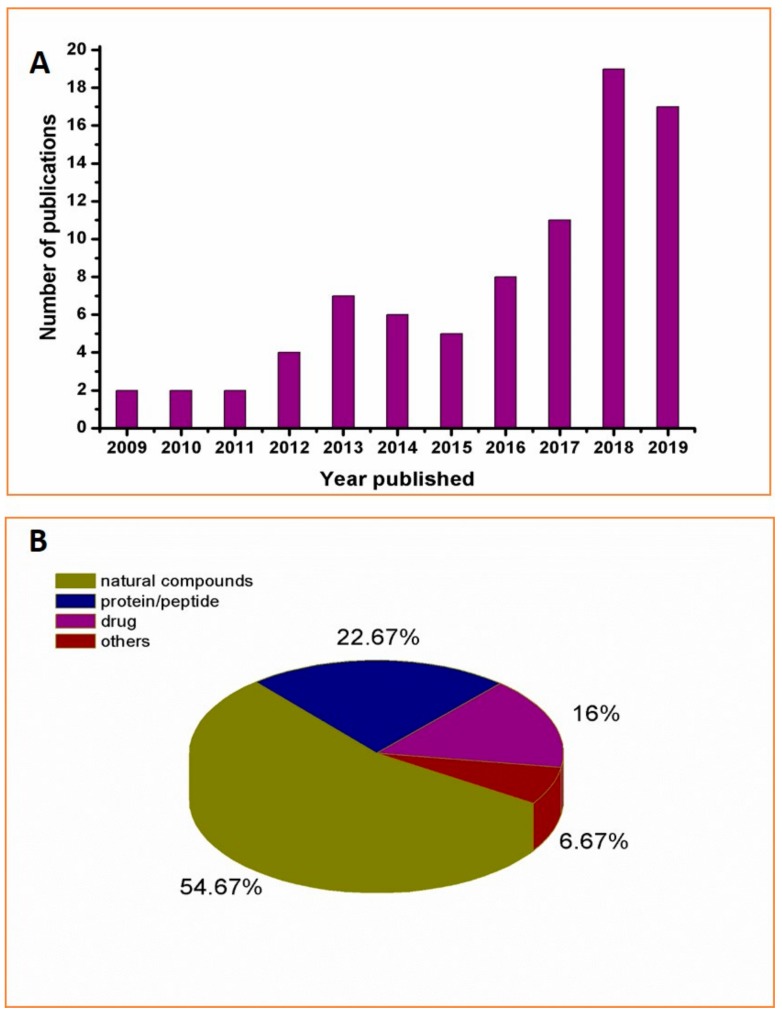
Number of publications searched containing “antimicrobial chitosan conjugate” keywords via ISI Web of Science (**A**); Classification and proportion of corresponding chitosan conjugates (**B**); Note: The data for 2019 are as of the end of November.

Since 2000, there have been sporadic reports on the antimicrobial activities of chitosan conjugates, but there was little research in this area throughout the 2000 s. It was not until 2010 that chitosan conjugates used for antimicrobials attracted increasing attention. In the past three years, related research has experienced explosive growth, as shown in Figure 1A. Moreover, it was found that the coupling strategy of chitosan conjugates is generally through the coupling of natural products such as polyphenols [45], organic acids [46], and proteins/polypeptides such as lysozyme [47] or through the coupling of commercially available antimicrobials such as gibberellin [38], and sulfadiazine [48] (Figure 1B). Due to the diversity of coupled active molecular structures, their corresponding coupling methods are also different. Below, we will discuss and summarize the coupling methods in detail.

### 2.1. Free Radical-Induced Conjugation

The free radical-induced conjugation method is commonly used in the synthesis of chitosan polyphenol conjugates due to its economical, convenient, and eco-friendly properties. Polyphenols are secondary metabolites of plants and are usually found in fruits, vegetables, teas and coffees. The most common phenolic substances are phenolic acids, flavonoids, stilbenes, and lignans [49]. These compounds are involved in various physiological activities including nutrient intake, protein synthesis, photosynthesis, etc. More importantly, it has been extensively proven that polyphenols possess good antimicrobial, antioxidant and other activities, which have been attracted increasing interest in recent years [50,51,52,53].

For the synthesis of conjugates between phenolic acids and chitosan, the free radical reaction is often initiated by the H_2_O_2_/VC system [45,54,55,56,57,58]. This system has several advantages. First, no toxic intermediates or products are generated during the reaction. Moreover, the reaction conditions are mild, and usually only need to be carried out at relatively lower temperatures, thereby reducing the possible decomposition of polyphenol active ingredients under higher temperature conditions. However, the diversity and complexity of the chitosan conjugate structure also put forward higher requirements for its structural characterization. In addition to conventional NMR, IR, and other methods, electron paramagnetic resonance (EPR) has also been demonstrated to play a key role in studying the mechanism of free radical grafting reaction of chitosan [59,60,61,62]. This is because EPR spectroscopy is useful for elucidating the species of free radicals present in a reaction and distinguishing carbon, nitrogen or oxygen-based free radicals.

**Figure 2:**
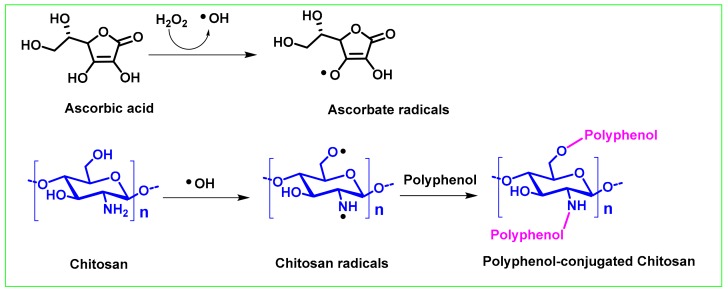
Proposed mechanism of synthesis of polyphenol chitosan conjugate by free radical induction reaction.

The proposed mechanism is shown in Figure 2. First, ascorbic acid reacts with hydrogen peroxide at room temperature to produce hydroxyl and ascorbate free radicals. Afterwards, the generated hydroxyl radical captures hydrogen atoms on the polysaccharide chain -OH, and -NH_2_ groups to activate the chitosan to form a chitosan radical. Finally, the polyphenol serves as an acceptor and reacts with the chitosan radical to form a chitosan polyphenol conjugate [33,57]. 

Since the degree of substitution (DS) of the conjugate is a key factor affecting its activity, many recent studies have focused on increasing the DS by optimizing the reaction conditions [54,55,57,58,63]. Some studies have shown that by increasing the ratio of polyphenols to chitosan in the critical range, the polyphenol content in the conjugate can be increased. However, beyond that range, excessive free polyphenol molecules may inhibit the progress of the reaction, resulting in a decrease in the coupling rate [54,63]. Therefore, although the radical reaction has advantages such as being green and economical, its low derivatization degree is an urgent problem to be solved, and thus other functionalization strategies such as chemical condensation, enzyme-assisted, and electrochemical methods have been gradually explored.

### 2.2. Carbodiimide Chemistry

The free amino and hydroxyl groups present in the chitosan molecule may undergo acylation and esterification reactions. Synthetic methods commonly used in such reactions include an acyl chloride method, a mixed acid anhydride method, an activated ester method, and a carbodiimide method. For example, İlyasoğlu and Guo reported the synthesis of soluble chitosan-caffeic acid conjugates. First, caffeic acid reacted with SOCl_2_ to form an acyl chloride, which then reacts with a free amino or hydroxyl group of chitosan to form an amide bond and an ester bond, respectively. The addition of dimethyl aminopyridine (DAMP) as a catalyst can promote the reaction of acyl chlorides and amino groups [31]. However, the disadvantage of this method is that acyl chloride is formed under acidic conditions and that many acid-sensitive groups cannot withstand it. To avoid degradation of chitosan and loss of its biological activity, crosslinking of biologically active molecules with chitosan should be carried out using mildly reactive reagents under mild conditions (e.g., near-neutral pH, room temperature, aqueous solution). Carbodiimide is an ideal reagent for satisfying the above reaction conditions and thus has been widely used in the synthesis of chitosan conjugates [32,38,46,64,65,66,67,68,69,70,71,72,73]. Currently, there are three main types of condensing agents: dicyclohexylcarbodiimide (DCC) and diisopropylcarbodiimide (DIC), and 1-(3-dimethylaminopropyl)-3-ethylcarbodiimide (EDC). The use of such a condensing agent generally requires the addition of an acylation catalyst or an activator including 4-*N,N*-lutidine (DMAP), 1-hydroxybenzotriazole (HOBt), N-hydroxysuccinimide (NHS) etc. The carbodiimide condensation reagent method is currently the most widely used method for forming an amide bond and is also widely used in the construction of ester bonds, macrolactams and lactones. In this method, the carboxyl component and the amino component are usually mixed, and the intermediate is directly reacted to form an amide bond without separation by the action of a condensation reagent. Thus, it is not necessary to prepare a carboxyl-activated intermediate such as an acyl halide, acid anhydride or activated ester in advance, which is not only simple and efficient, but can also effectively avoid some side reactions generated during separation and purification of the activated intermediate and storage. 

**Figure 3:**
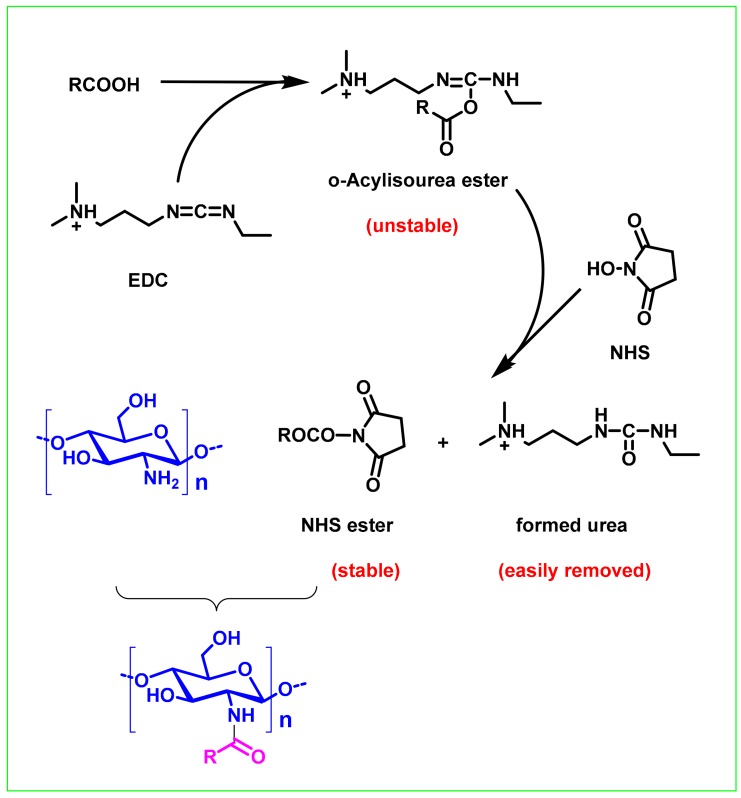
Proposed mechanism of synthesis of a chitosan conjugate by a carbodiimide based chemical coupling method.

The earliest carbodiimide condensation reagent used was *N*,*N*-dicyclohexylcarbodiimide (DCC) [74]. However, the dicyclohexylurea (DCU) formed by the reaction has limited solubility in most organic solvents and is difficult to remove. At present, the commonly used carbodiimide chemistry is the EDC/NHS system (Figure 3). The proposed mechanism of the reaction is as follows: First, the carboxylic acid group is activated by EDC to produce an O-acylisourea group; then it is converted into a more stable active NHS-activated carboxylic acid group, and finally coupled with the amino group of chitosan to form the chitosan conjugates [75]. One of the main characteristics of this process is that the urea formed after the reaction is water-soluble and can be easily washed off. Due to the mild reaction conditions and simple workup process, this approach has become one of the preferred methods for the synthesis of chitosan conjugates. However, the reaction selectivity of EDC is not very good, it can react with the amino groups of chitosan and hydroxyl groups. Therefore, functional group protection is required to ensure accurate synthesis of chitosan conjugates [73]. However, the reality is that most of the current studies have not adopted a strategy of functional group protection.

### 2.3. Coupling by Forming a Schiff Base

Among the diverse chitosan conjugates, imine-linked chitosan conjugates have recently received considerable attention. The primary amino group contained in the chitosan skeleton can easily undergo a condensation reaction with an acyl compound (aldehyde, ketone) to form a Schiff base [76,77,78,79,80,81]. This reaction can be performed in a green solvent such as water and ethanol, and the reaction conditions are mild and green. No toxic reagents are introduced during the reaction. More importantly, this reaction is a specific reaction between amino and acyl groups. During the reaction, no side reactions will occur with the free hydroxyl groups of chitosan. Therefore, the reaction has the advantages of good regioselectivity and environmental friendliness. It has been reported that galactose [82], curcumin [35], inulin [83], and caffeic acid [84] can be successfully coupled with chitosan to obtain the corresponding conjugates by this method. It should be noted that, considering the instability of the Schiff base, the chitosan Schiff base conjugate is usually reduced to a stable amino-substituted chitosan conjugate through sodium borohydride or sodium cyanoborohydride [37,85,86]. 

### 2.4. Functional Group Conversion Strategy

**Figure 4:**
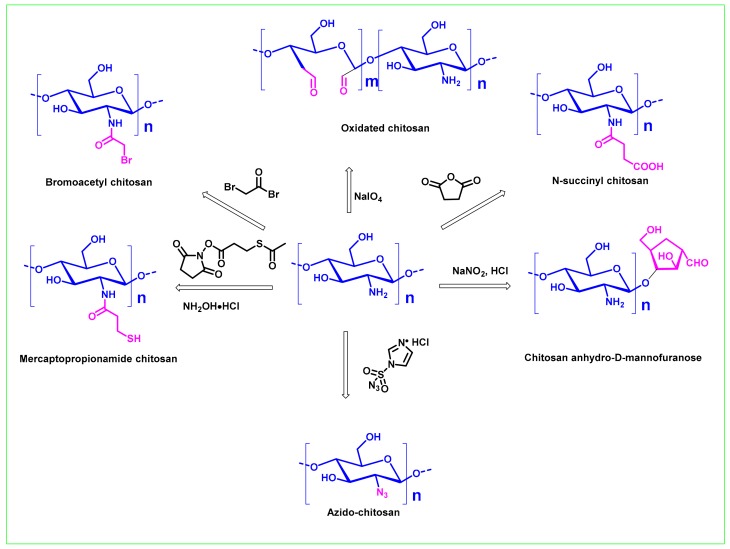
Functional group conversion strategies commonly used in the synthesis of chitosan conjugates.

Although the presence of hydroxyl and amino groups in the chitosan molecule can allow structural modification to be easily carried out, in terms of conjugates that result in chitosan conjugates with more structural diversity, it is very necessary to explore a functional group conversion strategy to introduce new reactive groups to the chitosan backbone. It has been demonstrated that the amino group of chitosan can be converted into azide group [87], substituted carboxyl group [88], substituted mercapto group [89], etc., and the hydroxyl group can be azidated [90], aminated [91,92], oxidized to an aldehyde [93] or carbonyl group [94], or further oxidized to a carboxyl group [95]. Figure 4 lists some common functional group conversion methods used in the preparation of common chitosan conjugates. For example, the 2,3-diols of chitosan can be oxidized by NaIO_4_ to form partially oxidized chitosan [94]. Chitosan anhydro-D-mannofuranose can be obtained by nitrous acid depolymerization of chitosan [93]. Recently, Barbosa et.al. developed a method for preparing azide chitosan using imidazole-1-sulfonyl azide hydrochloride, which avoided the use of unstable and explosive azide reagents such as azide ions and triflyl azides [96]. It should be pointed out that some functional group conversion strategies such as introducing spacer arms-bearing active groups into chitosan can not only increase the reaction site, but also reduce the crowding effect, increase the reactivity, reduce steric hindrance, and improve the coupling rate and other functions. The use of spacer arms can enhance the binding of ligands to polysaccharides and provide a variety of binding sites. To date, functionalized chitosan with new reactive groups such as aldehyde groups, carbonyl groups, carboxyl groups, and thiol groups have been used widely for conjugations [82,97].

### 2.5. Enzyme-Assisted Coupling Reaction

Biological enzymatic conjugation of chitosan is mainly based on the catalytic oxidation of biological enzymes, which stimulates the grafting compound (generally a substrate of laccase) to form a highly reactive intermediate. The enzymes commonly used to modify chitosan are polyphenol oxidases, including tyrosinase [98], peroxidase [99], and laccase [100,101,102]. The grafted bioactive substances are mostly phenolic compounds such as cinnamic acid, ferulic acid and lauric acid. 

**Figure 5:**
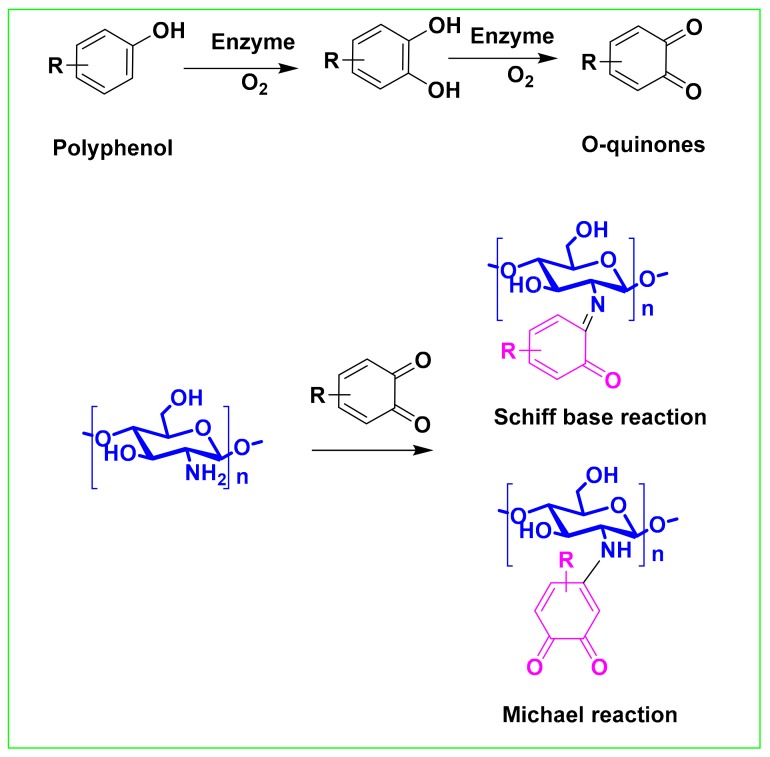
Proposed mechanism for the synthesis of polyphenol-chitosan conjugates through an enzyme-mediated strategy.

The enzyme catalysis reaction mechanism of chitosan and phenolic compounds is not clear at present, but the prevailing view is that laccase and tyrosinase are used to catalyze the conversion of phenolic compounds to quinone, which is more reactive. A non-enzymatic reaction that undergoes a Schiff base or a Michael addition reaction with chitosan generates a chitosan-phenolic conjugate (Figure 5) [103,104]. 

By catalysis with these biological enzymes, chitosan conjugates with new or better properties than the original chitosan can be obtained. This approach is expected to expand the application of chitosan in the pharmaceutical, cosmetics, and food industries. The biosynthesis method does not use any chemical reagents and has the advantages of good environmental compatibility, safety, and weak-side reactions. However, the method requires harsh reaction conditions. At the same time, the reaction will cause the hydroxyl group in the phenolic acid to be oxidized, which will reduce the activity of the synthetic product [105].

### 2.6. Other Methods

In the synthesis of chitosan conjugates, in addition to the above synthesis methods, other methods have also been reported in the literature. Examples include the Maillard reaction [106], acid-base salt formation reaction [36], and electrochemical reaction [107]. For instance, the Maillard reaction is commonly used for the coupling of xylan [108], polylysine [109], and lysozyme [47,106,110] with chitosan. The Maillard reaction is a non-enzymatic browning reaction, which occurs between the ε-amino group in a protein and the reducing carbonyl group in a polysaccharide upon heating. Generally, Maillard reactions follow a complex mechanism that is divided into three main phases (early, advanced, and final) [111]. Maillard-type protein-polysaccharide conjugates show potential applications because of their excellent and thermal stability, solubility and antimicrobial activity. Chitosan, coupled with lysozyme through the Maillard reaction, can also bring improved antibacterial activity and stability [110]. However, the Maillard reaction has a complex reaction mechanism, and the complexity of its conjugate structure poses challenges for precise structural analysis and repeatable synthesis.

Electrochemically assisted coupled reactions are also a method worthy of attention because of their environmental and safety advantages. For example, Kim et al. reported the successful fabrication of a chitosan-phenolic film via electrochemistry of catechol oxidation and putative chemistry [107]. However, so far, relatively few reports have investigated this type of method, and its reaction mechanism and procedure need to be further studied and optimized.

## 3. Physiochemical Properties, Antimicrobial Activities, and Potential Applications 

### 3.1. Physiochemical Properties and Antimicrobial Activities

As mentioned above, there are various types of coupling methods for chitosan conjugates. Active molecules can be covalently bonded to chitosan backbone or linked to the chitosan molecule through a linker. So how to choose a suitable coupling method, process, regents, and linker are crucial for subsequent activity research and practical application. Generally, the ideal conjugating approach should ensure a high yield of the conjugate, a uniform composition of the conjugate, a suitable binding ratio, maximum biological activity, convenient operation, and easy purification. More examples of chitosan conjugate systems were listed in Table 1.

With the construction of the chitosan coupling system, the physical and chemical properties of chitosan, including thermal stability, solubility, crystallinity, etc., will generally change accordingly: (1) Thermal stability. The thermal stability of the chitosan conjugate is closely related to polysaccharides and bioactive substances. Some studies have shown that the introduction of exogenous active substances can lead to a decrease in the thermal stability of chitosan, which may be due to the weakening of the strong intramolecular bonding in the chitosan chain and the obstruction of chitosan chain packing due to the coupling reaction [41,74,113]. This decrease in thermal stability usually does not affect the actual application of chitosan, because the structural modification only partially reduces the thermal stability of chitosan, and its conjugates still have high thermal stability (usually up to 200 °C) [113]. Even more exciting is that due to the decrease in the intramolecular force of chitosan, its solubility is improved instead [31]. (2) Solubility. Modification of chitosan often significantly improves its water solubility [25,31,114]. This is because the introduction of bioactive groups reduces the force of intramolecular hydrogen bonding interactions within chitosan. The steric hindrance is increased due to the aggregation of polysaccharide chains. In addition, small active molecules such as phenolic acid groups contain many hydroxyl groups to enhance the interaction between the conjugate and water. (3) Crystallinity. The coupling reaction often reduces the crystallinity of chitosan conjugates [69,74]. This may be due to the intra- and intermolecular hydrogen bonding interaction of chitosan being destroyed after the reaction, which is consistent with the abovementioned decrease in the thermal stability of chitosan. 

In addition to the changes in physicochemical properties brought about by the chitosan coupling strategy, more interesting is the improvement of the antimicrobial activity of chitosan. Although it has been demonstrated that chitosan displays broad-spectrum antimicrobial activities against gram-positive bacteria, gram-negative bacteria and fungi, it is true that relatively poor activity of chitosan hinders its practical application. Compared with chitosan itself, the chitosan conjugates can have improved the antimicrobial properties of chitosan, as well as the improved poor water solubility of chitosan, which allowing the conjugates can to be used in neutral environments. For example, Lee et al. reported the synthesis of chitosan–caffeic acid, chitosan–ferulic acid (CFA), and chitosan–sinapic acid conjugates with enhanced antimicrobial activity against Staphylococcus aureus and foodborne pathogens. Among the chitosan conjugates, the MIC values of the CFA is in range of 32–64 μg/mL, which is much lower than that of unmodified chitosan [54]. Wang et al. described the antimicrobial properties of different chitosan phenolic acids conjugates. It was found that all the chitosan conjugates exhibited significantly higher antimicrobial activities. Moreover, it was reported by Lee and Je that gallic acid conjugated chitosan displayed much better antibacterial activities than unmodified chitosan, of which the MIC value ranged from 16 to 64 μg/mL against the tested bacteria. Interestingly, it was thought that gallic acid in the chitosan conjugate does not exert major antibacterial activity, and there may be synergistic effects of phenolic acid and CS through conjugation [115]. Similar results have also been reported by two other labs [37,86], suggesting that the coupling of chitosan and streptomycin may play a synergistic effect. In general, chitosan conjugates generally have a broader spectrum and higher antimicrobial activity than chitosan. However, their antimicrobial properties can be affected by multiple factors such as strains, pH values, and degree of substitution, so it is necessary to further study the activity and mechanism. More antimicrobial data on chitosan conjugates can be found in Table 2.

### 3.2. Potential Applications 

Chitosan is favoured by scientific researchers for its wide potential application in different fields. After modification, the advantages of chitosan such as good biocompatibility and non-toxicity are maintained, and the water solubility and antimicrobial activity are enhanced (Table 3). In addition to the physiochemical parameters mentioned above, during modification of the chitosan structure, other physical and chemical properties such as viscosity, emulsification performance, and mechanical strength may also change accordingly, as shown in Table 3. As a result, the newly synthesized chitosan conjugate has many new properties that are different from that of chitosan.

**Figure 6:**
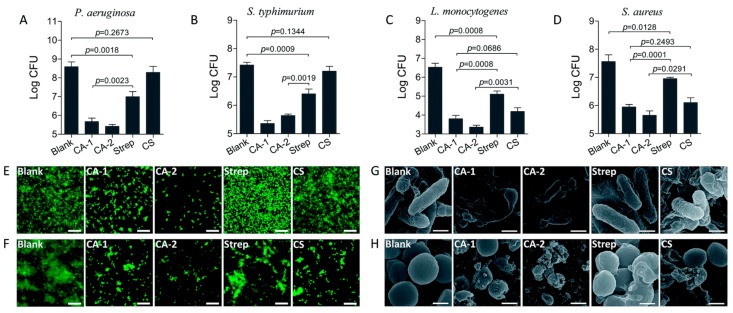
Effect of CA NPs on prefabricated biofilms formed by gram-negative and gram-positive organisms. Biofilm produced by *Pseudomonas aeruginosa* (**A**)*,* *Salmonella typhimurium* (**B**), *Listeria monocytogenes* (**C**), and *Staphylococcus* aureus (**D**) treated with CA NPs (250 mg/mL), CS (250 mg/mL), streptomycin (Strep, 50 mg/mL) for 24 h; After 24 h of treatment, pre-formed biofilm structures of Pseudomonas aeruginosa (**E**) and *Staphylococcus aureus* (**F**) under a fluorescence microscope (Scale bar represented for 10 μm); After 24 h of treatment, pre-formed biofilm structures of *Pseudomonas aeruginosa* (**G**) and *Staphylococcus aureus* (**H**) under a scanning electron microscopy (Scale bar represented for 400 nm). Reprinted with permission from [37].

The special properties of disinfection and promotion of wound healing of the chitosan conjugate make it very potential for development as an antimicrobial agent. The chitosan conjugates have shown great application prospects in artificial skin, wound dressing materials and antimicrobial surfaces. For example, Zhou et al. synthesized a class of chitosan LED209 conjugates based on chemical coupling method. Compared with chitosan, the conjugates displayed better solubility and higher selectivity for *MDR-E. coli.* Further studies have shown that the conjugates can maintain the characteristics of CS and LED 209, and prevent bacterial adhesion. Therefore, these findings provide a feasible strategy for the synthesis of multifunctional antibacterial agents [114]. Mu et al. successfully combined chitosan with streptomycin, thereby improving the ability of antibiotics to resist biofilms formed by gram-positive bacteria rather than gram-negative bacteria, and thus providing a new solution to the problem of antibiotic resistance. In this study, a robust nanoparticle was developed by introducing gold (Au) nanoparticles into a chitosan-streptomycin conjugate (CS), with the product called CA NPs. It was proved that the nanoparticles have a strong double-membrane destruction activity against gram-negative bacteria (Figure 6) [37].

Another antimicrobial application of chitosan conjugates is as a carrier for antibiotics. As a natural high-molecular-weight polymer, chitosan is widely used in drug delivery systems, especially anti-cancer drugs. In chitosan-drug combination systems, chitosan and drugs form conjugates, which can achieve targeted delivery and controlled release of drugs, thereby improving bioavailability and treatment efficacy. However, in terms of bacteriostatic applications, only a few studies have been conducted on coupling chitosan with antibacterial drugs for drug release [41,48]. The current research focus of chitosan conjugates is mainly focused on the coupling of chitosan with natural antibacterial substances and their applications as external antibacterial materials. For example, Xu et al. described the synthesis of a new type of thermo-responsive chitosan–catechol–pNIPAM wet adhesive conjugate. The synthesized chitosan conjugates have reversible sol-gel transition behaviour and thermally responsive wet adhesion. By taking advantage of these outstanding features, the conjugates can achieve controlled attachment/detachment behaviour on the skin through heating/cooling processes. Moreover, this material is expected to be used as an intelligent adhesive in various biomedical environments [67]. 

The film-forming properties of chitosan can also allow it to be applied to food packaging and preservation. In addition to serving as a protective barrier, the edible chitosan films can be used as a carrier for bioactive compounds to improve food quality. The combination of chitosan and different antimicrobial agents, such as organic acids, plant extracts, antibiotics, etc., can reduce food spoilage of pathogenic microorganisms and extend shelf life [45,63,121]. 

As mentioned above, chitosan conjugates have many excellent properties and have broad antimicrobial application prospects in the pharmaceutical and food industries. Chitosan conjugates, like chitosan, have inherent advantages in preventing wound infection and promoting wound healing due to their good biocompatibility and low toxicity. This greatly enhances the application value of chitosan derivatives and provides more options for preparing biocompatible and non-toxic antimicrobial agents.

## 4. Challenge and Limitations

### 4.1. How Can Chitosan Conjugates Be Properly Designdesigned and Synthesized to Ensure Their Effectiveness?

As mentioned above, there are many types of chitosan coupling and cross-linking methods, each with their own advantages and disadvantages. Determining how to properly design the chitosan conjugate and select the appropriate coupling method is a challenge in itself. When evaluating a conjugating method, the following relevant factors should be considered: (a) The effect of the coupling reaction, such as being a green synthesis method and having easy post-processing, the homogeneity of the composition of the conjugates; (b) The yield of the conjugation reaction and the degree of substitution (DS) of the conjugates; (c) The effect of the coupling process on the biological activity; and (d) Clear application objects and scope. Overall, the ideal conjugating method should ensure a high yield of the conjugate, a uniform conjugate composition, a suitable binding ratio, maximum biological activity, convenient operation, and easy purification; moreover, under the same conditions, the method should have good repeatability. In the preparation of the conjugate, it is also required that the prepared conjugate maintain the structural specificity of the original active substance; the conjugating method used should not significantly change the original structure or introduce toxic groups. 

The following points are specifically emphasized: It is generally believed that the higher the DS of the chitosan conjugate is, the stronger its antibacterial activity is [114]. However, due to the structure of the chitosan macromolecule and its potential steric hindrance, the coupling rate of chitosan is not high in many cases [33,57,63], which may bring the problem of the insufficient effective concentration of active ingredients in the body. Another very important issue is that macromolecular conjugates are first required to be able to provide quantitative or targeted sustained release of free biologically active drugs in vivo. To this end, the coupling bond that connects the drug to the carrier must be able to dissociate at a certain rate under a physiological environment. Moreover, the introduction of chemically linked fragments or reactive groups during the construction of the materials results in not all the chitosan-based conjugates exhibiting good biodegradability and compatibility, and conversely, even producing toxic side effects [112].

Therefore, an appropriate coupling method must be selected according to the purpose of the conjugate and the advantages and disadvantages of different methods must be weighed. Reasonable conjugate design is the premise to ensure its safety and effective therapeutic effect.

### 4.2. How Can the Structure-Activity Relationship and Mechanism of Action of the Chitosan Conjugates Be Clarified?

The antimicrobial activities of chitosan, as a class of biological macromolecules, are affected by many factors including molecular weight, degree of deacetylation, pH, etc. [122,123,124]. There are often large differences in antimicrobial activity in different studies, and some studies even obtained diametrically opposite results, which makes it a great challenge to accurately evaluate the structure-activity relationships. Therefore, although the introduction of active ingredients on the chitosan sugar chain improves its activity, it also brings more complexity to the structure and results in greater challenges determining the structure-activity relationship and antimicrobial mechanisms. In addition, the covalent bonding of the active ingredient to the chitosan carrier is usually a random process. Selecting the reaction site of the active ingredient in the sugar chain remains an uncontrollable problem. What is more important—How to clarify the functional differences between chitosan conjugates and chitosan and active ingredient mixtures? Is coupling necessary or redundant? Considering that chitosan conjugates are relatively complex systems that differ from traditional small molecules with a well-defined chemical structure, accurately characterizing their structure is a great challenge. The superposition of all the above factors brings great difficulties in studying the structure-activity relationships of chitosan conjugates. 

The antimicrobial mechanism of chitosan is still controversial. It may have different modes of action against gram-positive bacteria, gram-negative bacteria and fungi [122]. At present, the generally accepted view is that the electrostatic interactions between the protonated amino group of the chitosan molecule and the anionic surface of the pathogen under acidic conditions are the key to determining the chitosan antimicrobial mechanism. The introduction of active molecules can improve its antimicrobial properties as well as increase its water solubility and expand its application range. However, the diversity of the chitosan conjugate structure influences the complexity of its antimicrobial mechanism. Does the chitosan conjugate work as a system or does it work by slow-releasing active ingredients? What role does chitosan play in this process? Is it just a carrier? There are still many questions that need to be studied and answered.

## 5. Conclusions and Outlook

In the past decade, great progress has been made in the study of chitosan conjugates. Many chitosan conjugates with diverse structures and functions have been synthesized and show potential application prospects. However, at present, there is no clear mechanism for the antimicrobial effect of chitosan and its conjugates. Moreover, studies on chitosan conjugates are mostly focused on their physicochemical properties and in vitro antimicrobial effects, which delays their practical applications. Therefore, the progress and continuous development of chitosan conjugate research urgently require the multi-disciplinary and multi-collaborative collaboration of polymer chemists, medicinal chemists, and pharmaceutical scientists. It is believed that with the rapid development of cell biology, molecular biology, materials chemistry and nanotechnology, chitosan conjugates will play a greater role in antimicrobial therapy.

## Figures and Tables

**Table 1:** Representative chitosan conjugate systems.

Number	Sample	Active Molecule	Linker	Bond Type	References
1	Chitosan-tannic acid: Conjugate	tannic acid	No	Amide/ester	[33]
2	Gallic acid-chitosan conjugate	gallic acid	No	Amide/ester	[55]
3	Chitosan–hydroxycinnamic acid conjugates	hydroxycinnamic acid	No	Amide/ester	[54]
4	Gibberellin–chitosan conjugate	gibberellin	No	Amide	[38]
5	Chitosan-caffeic acid conjugates	caffeic acid	No	Amide	[31]
6	Curcuminconjugated chitosan	curcumin	No	Imino	[35]
7	Chitosan-isoniazid conjugates	isoniazid	epichlorohydrin	Hydrazo/hydrazide	[112]
8	Chitosan–thymine conjugate	thymine	bromoacetic acid	Amide	[74]
9	chitosan-PVC conjugates	PVC	bromoacetyl bromide	Alkylamino	[97]

**Table 2:** Antimicrobial activities of representative chitosan conjugates.

Sample	Microorganism	Antimicrobial Properties	References
Chitosan-Catechin Conjugate	*Bacillus subtilis*	MIC: 64 μg/mL (Conjugate); 128 μg/mL (chitosan)	[34]
Chitosan-Caffeic Acid Conjugate	*Staphylococcus aureus*	MIC: 8 μg/mL (Conjugate); 16 μg/m(chitosan)	[116]
Chitosan–hydroxycinnamic acid conjugates	*Bacillus subtilis*	MIC: 2 μg/mL (CFA (I)); 128 μg/mL(chitosan)	[54]
Chitosan thiolated conjugate	*E. coli*	MIC: 2.9 mg/mL (TCNAC); 4.1 mg/mL(chitosan)	[39]
Chitosan thiolated conjugate	*Bacillus subtilis*	MIC: 0.25 mg/mL (CA-g-CS); 2 mg/mL(chitosan)	[69]
chitosan–lauricacid conjugate	*Staphylococcus aureus*	Antibacterial rate: 95.6% (Ti-PDOP-Chi–2.5%LA)	[117]
Cinnamic acids conjugated chitosan	*Ralstonia solanacearum−5*	IC_50_: 0.23 mg/mL (CTS-g-CA)); 0.56 mg/mL(chitosan)	[118]
Chitosan gallic acid conjugate	*C. albicans*	Reduction of fungi: 70%(GA); 61%(chitosan)	[100]
Sulfadiazine—Chitosan Conjugates	*Listeria monocytogenes*	Inhibition rate: 100% (PEC Lm-SDZ); 58%(chitosan)	[48]
Polylysine–chitosanconjugates	*Beer yeast*	Inhibition zone: 1.36 cm (conjugate) 0.96 cm (chitosan)	[109]
Hydrocaffeic acid conjugated chitosan	*S. epidermidis*	Enhanced antimicrobial activity compared to pure chitosan.	[119]

**Table 3:** Physicochemical properties, bio-functions, cytoxicity and potential applications of the typical chitosan conjugates *.

Sample	Potential Applications	Physiochemical Properties	Bio-Functions	Cytotoxicity	References
LED 209 conjugated chitosan	Antimicrobial and anti-adhesion material	Increasing solubility with increase in DS	Highly selective activity and anti-adhesion activity against MDR-*E. coli*	Minor cytotoxicity to mammalian cells.	[114]
Galabiose-chitosan Conjugate	Anti-adhesion agents	Showed good solubility in neutral water (1.0 mg/mL)	The highest inhibitory effect at the DP of 1839 (MIC:1.7 nM)	n.d	[120]
Gibberellin–chitosan	Fungicide release	Good water solubility and stability	n.d	n.d	[38]
Dhvar-5-chitosan conjugate	Antimicrobial surfaces	lower viscosity values	Displayed bactericidal effect.	No cytotoxic potential.	[96]
Curcumin conjugated chitosan	Anti-skin infection agent	n.d	Be effective against *E. coli* and *S. Auerus*	Cyto and hemo-compatible	[35]
Inulin–LCS Conjugate	Anti-biofilm reagent	Good water solubility	Showed similar biofilm eradication with florfenicol at 500 μg/mL	Low cellular toxicity to mammalian	[83]
Gallic acid-grafted-chitosan	Biomaterials in food packaging	An increase in water solubility; exhibited darker appearance and weaker transmittance	Significantly enhanced antibacterial ability	n.d	[72]
Gallic acid grafted chitin-glucan complex	Biomedical areas	n.d	Completely inhibited the growth of *Bacillus subtilis* and *Escherichia coli*	Non-hazardous and biocompatible	
Chitosan–hydroxycinnamic acid conjugates	Food and pharmaceutical industries	n.d	Exhibited better antimicrobial activity than chitosan	No cytotoxic activity	[56]
Chitosan–lysozyme conjugates	Ingredient with emulsifying properties	Greatly improved solubility and emulsion stability	Enhanced bactericidal action against *Escherichia coil* K-12,	n.d	[54]
lysozyme-chitosan oligosaccharide conjugates	Refractory infection drugs	n.d	Exhibited antibacterial activity and low drug resistance	Low hemolytic activity	[110]
Cefuroxime conjugated chitosan	Anti-chronic wound infection drugs	Decrease in the rate of swelling and degradation rate	Showed an efficient antibacterial activity over a longer period.	Have good blood compatibility	[106]
Chitosan-phenolic acid conjugates	Food preservatives	Improved water solubility	Showed broad spectrum antibacterial activity	n.d	[42]
Chitosan-isoniazid conjugates	Antituberculosis drugs	Enhanced solubility under physi-ological conditions	Comparable or slightly higher minimum inhibitory concentration for conjugates than for INH itself	Reduced biodegradability and decreased toxicity	[121]
Proanthocyanidin-chitosan conjugate	Food nutraceutical, and Biomedicine filed	Had lower crystallinity and thermal stability than chitosan	Showed bacterial strain-depended behavior in the antibacterial activity compared with chitosan	n.d	[112]

*: n.d.—not determined.
